# CapsTM: capsule network for Chinese medical text matching

**DOI:** 10.1186/s12911-021-01442-9

**Published:** 2021-07-30

**Authors:** Xiaoming Yu, Yedan Shen, Yuan Ni, Xiaowei Huang, Xiaolong Wang, Qingcai Chen, Buzhou Tang

**Affiliations:** 1 School of Political Science and Public Management, WuHan University, Wuhan, China; 2 Department of Computer Science, Harbin Institute of Technology, Shenzhen, China; 3 PingAn Health Technology Ltd, Shenzhen, China; 4 Peng Cheng Laboratory, Shenzhen, China

**Keywords:** Text matching, Deep learning, Capsule network, Chinese medical question matching

## Abstract

**Background:**

Text Matching (TM) is a fundamental task of natural language processing widely used in many application systems such as information retrieval, automatic question answering, machine translation, dialogue system, reading comprehension, etc. In recent years, a large number of deep learning neural networks have been applied to TM, and have refreshed benchmarks of TM repeatedly. Among the deep learning neural networks, convolutional neural network (CNN) is one of the most popular networks, which suffers from difficulties in dealing with small samples and keeping relative structures of features. In this paper, we propose a novel deep learning architecture based on capsule network for TM, called CapsTM, where capsule network is a new type of neural network architecture proposed to address some of the short comings of CNN and shows great potential in many tasks.

**Methods:**

CapsTM is a five-layer neural network, including an input layer, a representation layer, an aggregation layer, a capsule layer and a prediction layer. In CapsTM, two pieces of text are first individually converted into sequences of embeddings and are further transformed by a highway network in the input layer. Then, Bidirectional Long Short-Term Memory (BiLSTM) is used to represent each piece of text and attention-based interaction matrix is used to represent interactive information of the two pieces of text in the representation layer. Subsequently, the two kinds of representations are fused together by BiLSTM in the aggregation layer, and are further represented with capsules (vectors) in the capsule layer. Finally, the prediction layer is a connected network used for classification. CapsTM is an extension of ESIM by adding a capsule layer before the prediction layer.

**Results:**

We construct a corpus of Chinese medical question matching, which contains 36,360 question pairs. This corpus is randomly split into three parts: a training set of 32,360 question pairs, a development set of 2000 question pairs and a test set of 2000 question pairs. On this corpus, we conduct a series of experiments to evaluate the proposed CapsTM and compare it with other state-of-the-art methods. CapsTM achieves the highest F-score of 0.8666.

**Conclusion:**

The experimental results demonstrate that CapsTM is effective for Chinese medical question matching and outperforms other state-of-the-art methods for comparison.

## Background

Text matching (TM), which aims to judge whether two pieces of text, including sentences, questions, etc., are equal or match in semantic space, is a key component of many application systems such as information retrieval, automatic question answering, machine translation, dialogue system and reading comprehension. It is usually recognized as a classification problem where the input is a pair of pieces of text and the output is a label to indicate the two pieces of text match (denoted by 1) or not (denoted by 0).

In recent years, a large number of deep learning neural networks, such as Enhanced Sequential Inference Model (ESIM) [1], Attention-based Convolutional Neural Network (ABCNN) [2], Bilateral Multi-Perspective Matching (BIMPM) [3], Directional Self-Attention Network (DISAN) [4], Densely-connected co-attentive Recurrent Neural Network (DRCN) [5], Decomposable Attention Model (DECOMP) [6] and Bidirectional Encoder Representations from Transformers (BERT) [7], have been proposed for TM, and have achieved state-of-the-art performance on lots of benchmark datasets. Therefore, deep learning neural networks have become the mainstream machine learning methods for TM. Among these deep learning neural networks, convolutional neural network (CNN) is one of the most popular basic networks for TM. However, it suffers from difficulties in dealing with small samples and keeping relative structures of features. In this paper, we propose a novel deep learning architecture based on capsule network for TM, called CapsTM, where capsule network [8] is a new type of neural network architecture proposed to address some of the short comings of CNN. CapsTM is a five-layer neural network composed of an input layer, a representation layer, an aggregation layer, a capsule layer and a prediction layer. In this neural network, two pieces of text are first individually converted into embeddings sequences and are further transformed by a highway network in the input layer. Then, Bidirectional Long Short-Term Memory (BiLSTM) is used to represent each piece of text and attention-based interaction matrix is used to represent interactive information of the two pieces of text in the representation layer. Subsequently, the two kinds of representations are fused together by BiLSTM in the aggregation layer, and are further represented with capsules (vectors) in the capsule layer. Finally, the prediction layer is a connected network used for classification. CapsTM is an extension of ESIM by adding a capsule layer before the prediction layer. We apply CapsTM to Chinese medical question matching and achieve considerable performance. Experiments conducted on a manually annotated corpus regarding Chinese question matching show that CapsTM outperforms six state-of-the-art neural networks, that is, ESIM [1], ABCNN [2], BIMPM [3], DISAN [4], DRCN [5], DECOMP [6] and BERT [7].

The contributions of this work are: (1) investigating Chinese medical question matching comprehensively from corpus construction to methods; (2) proposing a novel method based on capsule network for Chinese medical question matching, which outperforms other state-of-the-art methods for text matching.

## Related work

In recent years, deep learning methods have become mainstream for text matching, and many deep neural networks have been proposed. Most of deep neural networks are based on Siamese network [9] which aims to represent two pieces of text by the same structure. The representative neural networks are DSSM (deep structured semantic models) proposed by Huang et al. [10] and ARC-I/ARC-II proposed by Hu et al. [11]. DSSM first uses multi-layer fully connected neural network to represent two pieces of text, then computes their cosine similarity, and finally makes a prediction. ARC-I/ACR-II uses a CNN-based architecture to model both text semantic information and interactive information between two pieces of text.

By introducing new neural networks and techniques, many variants of DSSM and ARC-I/ARC-II have been proposed. Shen et al. presented CDSSM by replacing multi-layer fully connected neural network by CNN [12]. Palangi et al. developed LSTM-DSSM using LSTM instead of multi-layer fully connected neural network [13]. Yin et al. introduced attention mechanism into ARC-I/ARC-II and proposed attention-based CNN (ABCNN) [2]. Chen et al. proposed an enhanced LSTM for text inference, ESIM, which first used BiLSTM to represent text semantic information and attention matrix to represent interactive information between two pieces of text, and then fused the two kinds of information via BiLSTM and pooling. Wang et al. adopted the same architecture of ESIM with four kinds of attention matrices, called BIMPM [3]. DISAN is a light-weight neural net proposed to learn sentence embedding, based solely on a directional self-attention with temporal order encoded, followed by a multi-dimensional attention without any recurrent neural network/CNN structure [4]. DRCN is a densely-connected co-attentive recurrent neural network proposed by Seonhoon Kim et al. [5], each layer of which uses concatenated information of attentive features as well as hidden features of all the preceding recurrent layers to preserve the original and the co-attentive feature information from the bottommost word embedding layer to the uppermost recurrent layer. Ankur et al. proposed a simple neural architecture for natural language inference, called DECOMP [6], which uses attention to decompose the problem into subproblems that can be solved separately. BERT is a language representation model proposed by Jacob et al. [7], which is designed to pre-train deep bidirectional representations from unlabeled text by jointly conditioning on both left and right context in all layers. Capsule network [8] as a new type of neural network architecture proposed to address some of the short comings of CNN has showed great potential in image classification.

## Methods

**Fig. 1:**
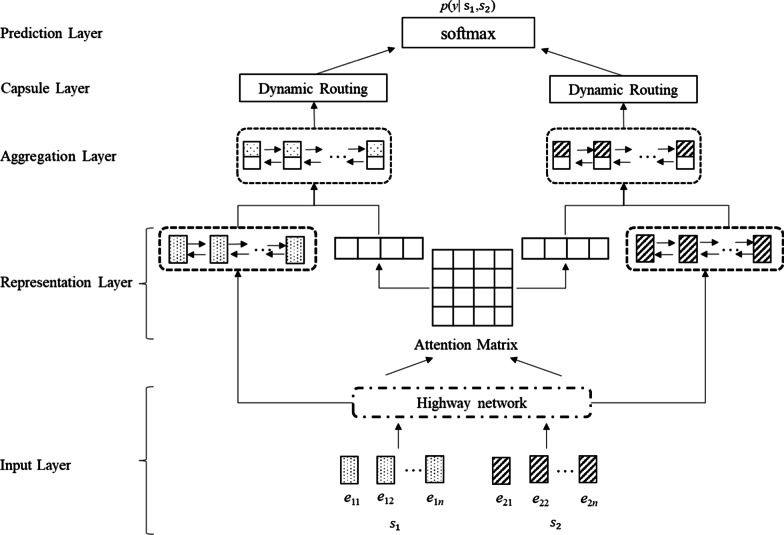
Architecture of CapsTM

Formally, the task of TM is to find the most possible label *y* (0-not match or 1- match) of the given pair of pieces of text (*s*_1_, *s*_2_), where *s*_1_ = w_11_w_12_…*w*_1*n*_ and *s*_2_ = *w*_21_*w*_22_…*w*_2*n*_ (*w*_*ij*_, the *j*-th word of *s*_*i*_ for *i* = 1, 2 and *j* = 1, 2, …, *n*) are two pieces of text of the same length after preprocessing that extends all pieces of text to the same length by appending dummy tokens. Figure 1 shows the overview architecture of CapsTM, which consists of an input layer, a representation layer, an aggregation layer, a capsule layer and a prediction layer. All these layers are presented in the following sections in detail.

### Input layer

For the given pair of pieces of text (*s*_1_, *s*_2_), the input layer first converts each piece of text into embeddings leant from large-scale unlabeled data by word2vec [14] or BERT [7], denoted by *e*_1_ for *s*_1_ and *e*_2_ for *s*_2_, and then further makes a transformation to the embeddings using highway network as follows:

1 $$\widehat{{e_{i} }} = \tan \;h(w_{f} e_{i} + b_{f} ),$$

2 $$g = sigmoid\left( {w_{g} \widehat{{e_{i} }} + b_{g} } \right),$$

3 $$e_{i}^{{\prime }} = g\widehat{{e_{i} }} + (1 - g)e_{i} ,$$

where *i* = 1,2, *w*_*f*_ and *w*_*g*_ are weight vectors, and *b*_*f*_ and *b*_*g*_ are bias vectors.

### Representation layer

In the representation layer, two types of information are extracted: (1) information of each piece of text; (2) interactive information of the two pieces of text. We utilize BiLSTM to extract the first type of information (Eq. 4) and attention-based interaction matrix to extract the second type of information (Eqs. 5–8) as follows:

4 $$h_{i} = BiLSTM\left( {e_{i}^{{\prime }} } \right),$$

5 $$sim_{ks} = e_{1k}^{{\prime }} .e_{2s}^{{\prime }} ,$$

6 $$a_{ks} = \frac{{sim_{ks} }}{{\mathop \sum \nolimits_{s} sim_{ks} }},$$

7 $$\widehat{{a_{1k} }} = \mathop \sum \limits_{s} a_{ks} e_{s} ,$$

8 $$\widehat{{a_{2k} }} = \mathop \sum \limits_{s} a_{sk} e_{s} ,$$

where $h_{i}$ is the concatenation of the last hidden states from forward and backward directions of BiLSTM, $e_{ij}^{^{\prime}}$ is the *j*-th vector of $e_{i}^{^{\prime}}$ (*i* = 1,2) corresponding to *w*_*ij*_. For the detailed information about BiLSTM, please refer to reference [1].

Finally, $h_{i}$ and $\widehat{{a_{i} }}$ are concatenated to form the representation of *s*_*i*_ for *i* = 1, 2, that is $c_{i} = \left[ {h_{i} :s_{i} } \right]$.

### Aggregation layer

This layer is employed to aggregate the representations of the two pieces of text using BiLSTM as follows:

9 $$S_{1}^{c} = BiLSTM\left( {c_{11} ,c_{12} , \ldots ,c_{1n} } \right),\quad S_{2}^{c} = BiLSTM\left( {c_{21} ,c_{22} , \ldots ,c_{2n} } \right),$$

where $S_{1}^{c}$ and $S_{2}^{c}$ are the concatenations of the last hidden states from forward and backward directions of BiLSTM for the two pieces of text.

### Capsule layer

**Fig. 2:**
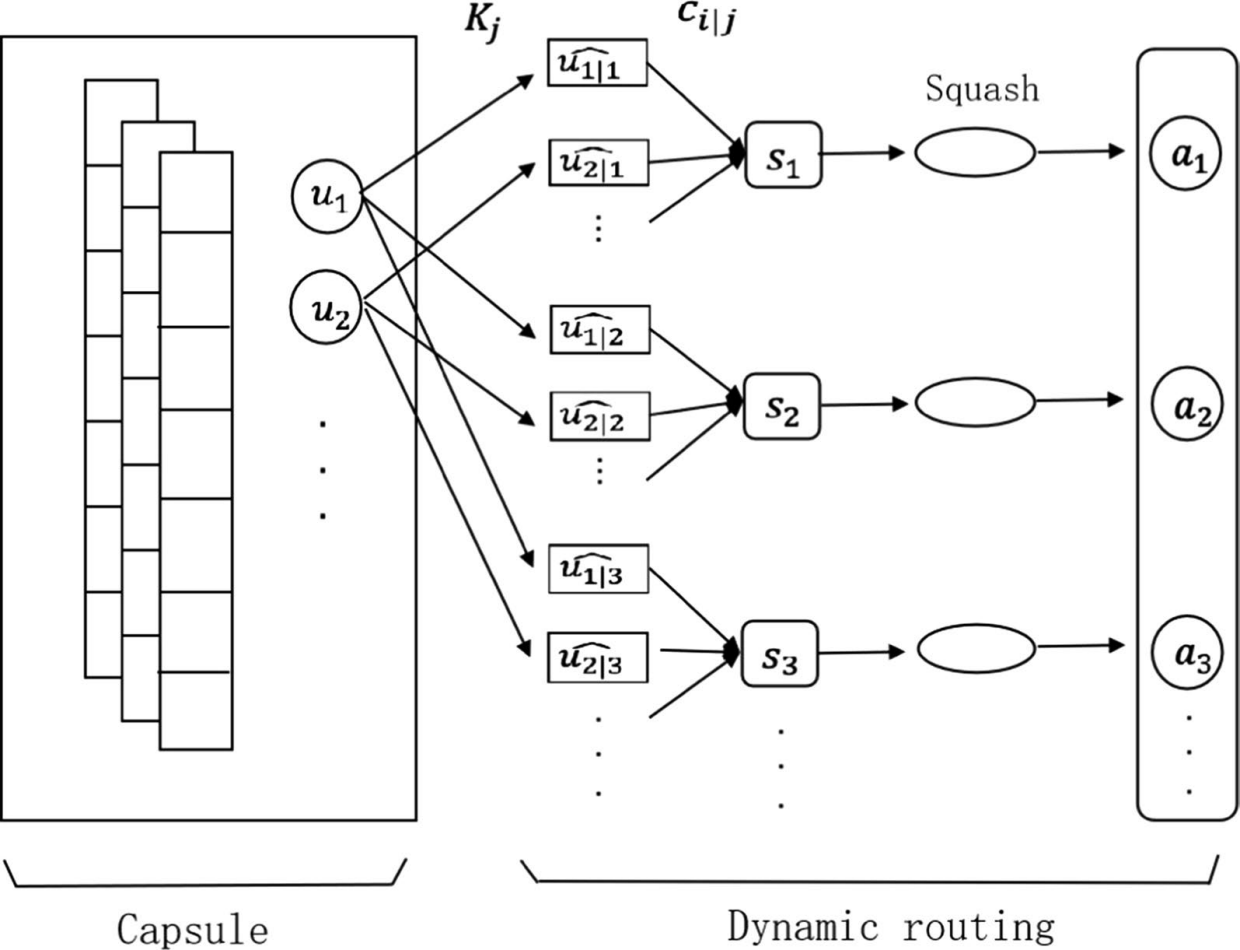
Architecture of capsule network

Capsule network (as shown in Fig. 2) adopts the dynamic routing algorithm (as shown in Table 1) to process the text representations from the aggregation layer iteratively. Firstly, *J d*-dimensional capsule networks are initialized. For each capsule, convolution operation is applied to $S_{1}^{c}$ and $S_{2}^{c}$:

10 $$F_{ij} = S_{i}^{c} .T_{j} + b_{j} (i = 1,2),$$

where $F_{ij}$ is the feature vector obtain from the *j*-th convolution kernel $T_{j}$ for *s*_*i*_, and $b_{j}$ is the bias vector for $T_{j}$.

**Table 1:** Dynamic routing algorithm

Dynamic routing algorithm
Initialize parameter $$b_{i|j}^{0} = 0$$
**for** *r* from 1 to *T* **do**
$$c_{i|j}^{r} = softmax\left( {b_{i|j}^{r - 1} } \right)$$
$$s_{j}^{r} = \mathop \sum \limits_{i} c_{i|j}^{r} \widehat{{u_{i|j} }}$$
$$d_{j}^{r} = Squash\left( {s_{j}^{r} } \right) = \frac{{\parallel s_{j} \parallel^{2} }}{{1 + \parallel s_{j} \parallel }}\frac{{s_{j} }}{{\parallel s_{j} \parallel^{2} }}$$
$$b_{i|j}^{r} = b_{i|j}^{r - 1} + d_{j}^{r} u_{i|j}$$
**return** $$d_{{\text{j}}}^{{\text{T}}}$$

Suppose that there are *I* convolution kernels, we can obtain *I*-channel feature vectors for *s*_*i*_:

11 $$U_{i} = \left[ {F_{i1} ,F_{i2} , \ldots ,F_{iI} } \right]$$

The generated *feature* vectors are then input into the capsule layer, which uses vector instead of scalar to save the instanced parameters of each feature. It can not only represent the intensity of activation, but also record some details of instanced part in the input. For each channel feature vector *u*_*i*_ in $U_{1}$ and $U_{2}$ (i.e., *u*_*j*_ = *F*_1*j*_ for *U*_1_ and *F*_2*j*_ for *U*_2_), convolution kernel $K_{j}$ (*j* = 1, …, *k*) is used to generate $u_{i|j} \in R^{d}$ for the *j*-th capsule using the following operation:

12 $$u_{i|j} = g(K_{j} .u_{i} + b),$$

where $g$ is a *nonlinear* activation function and $b$ is a bias vector. The *k* channels can be reconstituted to $\widehat{{u_{i} }}$:

13 $$\widehat{{u_{i} }} = \left[ {u_{i|1} ,u_{i|2} , \ldots ,u_{i|k} } \right]$$

Then, the dynamic routing algorithm (as shown in Table 1) is applied to generate capsules of the next layer. This process actually *replaces* the pooling operation that discards location information. At the beginning of the dynamic routing algorithm, the same weight is assigned to each location $c_{i|j}^{r}$ like the average pooling operation. After the first iteration, the weight of each location is updated according to the similarity between $c_{i|j}^{r}$ and $\widehat{{u_{i|j} }}$. The weight of each position is stable after iterating *T* times.

Finally, each piece of text *s*_*i*_ is represented by the outputs of all capsule networks:

14 $$C_{i} = \left[ {d_{1}^{T} ,d_{2}^{T} , \ldots ,d_{J}^{T} } \right]$$

### Prediction layer

The prediction layer is a fully connected network using the sigmod activation function for prediction. Following the previous work for TM [1–3], we use the following vector as the input of the prediction layer and the cross-entropy loss as the classification loss:

15 $$C = \left[ {C_{1} ,C_{2} ,C_{1} - C_{2} ,cos\left( {C_{1} ,C_{2} } \right)} \right]$$

## Experiments

### Dataset

We ask two medical experts to annotate a corpus of Chinese medical question matching, which contains 36,360 question pairs. This corpus is randomly split into three parts: a training set of 32,360 question pairs, a development set of 2000 question pairs and a test set of 2000 question pairs. The distributions of positive samples and negative samples in each dataset are listed in Table 2 in detail. Here, positive samples are the medical question pairs of the same meaning or intent, while negative samples are the medical question pairs of different meaning or intent.

**Table 2:** Distributions of positive samples and negative samples in the corpus used in this study

Dataset	Training		Development		Test	
	Positive	Negative	Positive	Negative	Positive	Negative
Number of smaples	12,610	19,750	801	1199	798	1202

### Experiment settings

We compare CapsTM with the following state-of-the-art deep learning neural networks: ESIM [1], ABCNN [2], BIMPM [3], DISAN [4], DRCN [5], DECOMP [6] and BERT [7]. All hyperparameters used in our experiments are shown in Table 3.

**Table 3:** Hyperparameters used in our experiments

Hyperparameter	Value	Hyperparameter (BERT)	Value
Embedding size	300	Embedding size	768
#Hidden states in BiLSTM	100	#Hidden states in BiLSTM	384
#Capsules	6	#Capsules	6
#Dimension of capsules	50	#Dimension of capsules	50
#Iterations	3	#Iterations	3
Learning rate	0.001	Learning rate	0.001
Dropout rate	0.5	Dropout rate	0.9
Activation function	ReLU	Activation function	ReLU
Batch size	32	Batch size	32

All Chinese character embeddings are pretrained by word2vec (https://code.google.com/p/word2vec/) and BERT (https://github.com/google-research/bert) on a large-scaled Chinese medical corpus. All model parameters are optimized on corresponding development sets. All the methods are implemented with Tensorflow 1.10.0, and all models are trained on machines with NVIDIA GeForce GTX 1080ti GPU. The performance of models is measured by precision (P), recall (R) and F-score.

## Results and discussion

As shown in Table 4 where all the highest values in each type are highlighted in bold, when using Chinese character embeddings initialized by word2vec, CapsTM(word2vec) achieves an F-score of 0.8432, and outperforms other state-of-the-art neural networks except BERT. The difference ranges from 0.65 to 3.85% in F-score. When using Chinese character embeddings initialized by BERT, the F-score of CapsTM(BERT) increases to 0.8666, which is higher than that of BERT by 0.2%. Compared to ESIM, CapsTM(word2vec) is significantly better with an improvement of 1.49% in F-score, indicating that the capsule layer added is effective.

**Table 4:** Comparison of CapsTM and other-state-of-the-art methods

Model	F-score	Precision	Recall
ESIM	0.8283	0.8156	0.8413
ABCNN	0.8144	0.8165	0.8123
BIMPM	0.8367	0.8190	0.8555
DISAN	0.8326	0.7800	**0.8930**
DRCN	0.8047	0.7877	0.8224
DECOMP	0.7951	0.7410	0.8577
**CapsTM (word2vec)**	**0.8432**	**0.8364**	0.8501
BERT	0.8646	0.8603	**0.8689**
**CapsTM (BERT)**	**0.8666**	**0.8655**	0.8677

In addition to investigate the effect of the attention mechanism used in the representation layer and the dynamic routing algorithm used in the capsule layer, we conduct ablation study on CapsTM. The results are shown in Table 5, where all the highest values in each type are highlighted in bold and w/o denotes “without”. When attention is removed or routing replaced by max pooling or mean pooling, the F-score of CapsTM drops. In the case of CapsTM(word2vec), the F-score decreases by at least 0.71% because of replacing routing by pooling and 2.16% caused by removing attention.

**Table 5:** Ablation study on CapsTM

Model	F1	Precision	Recall
CapsTM (word2vec)	**0.8432**	0.8364	**0.8501**
w/o routing (max)	0.8361	**0.8503**	0.8224
w/o routing (mean)	0.8295	0.8380	0.8212
w/o attention	0.8216	0.8005	0.8438
CapsTM (BERT)	**0.8666**	0.8655	0.8677
w/o routing (max)	0.8630	0.8635	0.8624
w/o routing (mean)	0.8646	**0.8680**	0.8613
w/o attention	0.8662	0.8641	**0.8683**

**Fig. 3:**
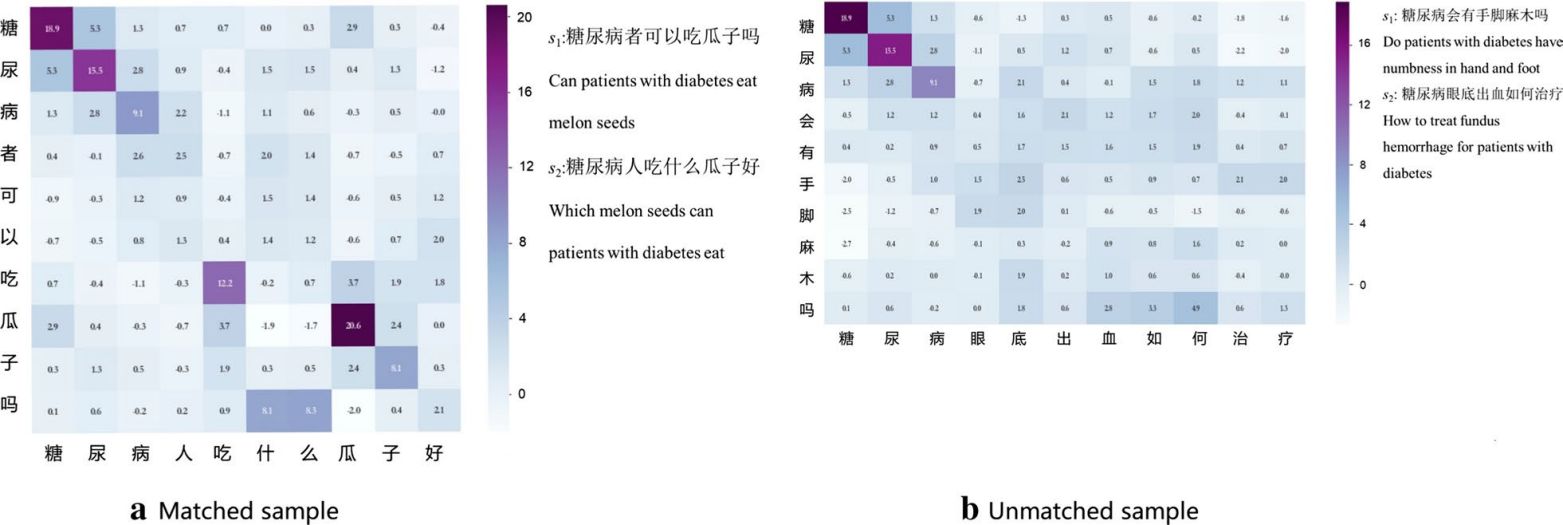
Visualization samples of the attention mechanism in the representation layer

Furthermore, we check the attention matrices of some samples and find that the attention mechanism can depict semantic similarities between words in question pairs. Figure 3 gives examples of a matched question pair and an unmatched question pair, where the darker the color is, the more semantically similar the question pair is.

There are also some errors in CapsTM. These errors mainly fall into the following categories: (1) question pairs of the same type with different topics are usually wrongly classified into 1. For example, “乙肝疫苗有效期为多久 (How long is the validation period of hepatitis B vaccine)” and “乙肝表面抗体能持续多久 (How long does hepatitis B antibody last)” are wrongly classified into 1. (2) the answer to one question covers the answer to another, but they are not the same. For example, the answer to “乙肝高血压如何用药 (How to take medicine for patients with hepatitis B)” should be included in the answer to “有乙肝病要如何控制高血压 (How to control hypertension of patients with hepatitis B)”, but we cannot answer the former question using the answer to the latter question directly. It is because that medication is only one type of treatments for hypertension of patients with hepatitis B. If we have a complete clinical knowledge graph, this problem may be solved. Therefore, for further improvement, we will investigate how to integrate clinical knowledge graph into existing state-of-the-art deep neural networks in the future.

## Conclusion

In this paper, we propose a novel five-layer neural network based on capsule network for Chinese medical TM, called CapsTM. Experiments on a manually annotated corpus shows that CapsTM outperforms other compared state-of-the-art neural networks. CapsTM can also have potential to be applied to TM in other domains.

## Data Availability

Not applicable.

## Abbreviations

TM: Text Matching
CNN: Convolutional neural network
CapsTM: Capsule Network for Chinese Medical Text Matching
BiLSTM: Bidirectional Long Short-Term Memory
ESIM: Enhanced Sequential Inference Model
ABCNN: Attention-Based Convolutional Neural Network
BIMPM: Bilateral Multi-Perspective Matching
DRCN: Densely-connectied Co-attentive Recurrent Neural Network
DECOMP: Decomposable Attention Model
BERT: Bidirectional Encoder Representations from Transformers
DSSM: Deep Structured Semantic Model
